# A 20-Year Spatio-Temporal Analysis of Gender Workforce in Medical Departments of Psychiatry/Psychotherapy and Neurology in Germany

**DOI:** 10.3390/jcm15145608

**Published:** 2026-07-17

**Authors:** Lisa Jörgens, Lena Löffler, Stefanie Mache, Doris Klingelhöfer, Isabelle Marie Kramer, Dörthe Brüggmann

**Affiliations:** 1 Department of Prevention, The Institute of Occupational, Social and Environmental Medicine, Goethe-University Frankfurt, 60590 Frankfurt, Germany; 2 Institute for Occupational and Maritime Medicine (ZfAM), University Medical Center Hamburg-Eppendorf (UKE), 20459 Hamburg, Germany

**Keywords:** gender disparities, leadership gap, workforce diversity, career advancement, career barriers, academic medicine

## Abstract

**Background:** Although almost two-thirds of medical students in Germany are female, medical leadership positions remain predominantly held by men. This study investigated gender-specific career progression in psychiatry/psychotherapy and neurology, two closely related clinical specialties, using spatio-temporal analyses over a 20-year period. **Methods:** The study analyzed five time points (2004, 2009, 2014, 2019, and 2024) to evaluate the temporal and spatial distribution of gender representation across Germany. Data on medical students, hospital-based specialists in psychiatry/psychotherapy and neurology, and department chairs were obtained from the Federal Statistical Office, the Federal Chamber of Physicians, and the medical faculties of 38 universities. Female-to-male ratios (f:m) were calculated for each career stage and applied to the established academic Brüggmann-Groneberg (BG) Index and to a newly developed clinical leadership version introduced in this study (BGclin Index). **Results:** In 2024, 64.9% of German medical students were female. Gender parity was reached among hospital-based specialists in psychiatry/psychotherapy and neurology, with f:m ratios increasing from 0.71 (2004) to 1.17 (2024) and from 0.40 (2004) to 1.00 (2024), respectively. Women remained markedly underrepresented in clinical leadership roles (2024 psychiatry/psychotherapy: 0.48; neurology: 0.23) and among department chairs (2024 psychiatry/psychotherapy: 0.15; neurology: 0.14). BG Indices reflected these disparities, with the highest academic BG Index observed in psychiatry/psychotherapy in 2019 (0.112) and in neurology in 2019 (0.091), while BGclin values remained higher, around 0.199 in psychiatry/psychotherapy and around 0.120 in neurology. Spatial analysis revealed substantial regional variation across Germany, with persistent gender disparities across federal states. **Conclusions:** Despite achieving gender parity at the specialist level, substantial gender disparities persist in both academic and clinical leadership in the examined specialties. The BG Indices highlighted a sustained disparity in women’s advancement, which was more pronounced in neurology than in psychiatry/psychotherapy.

## 1. Introduction

To this day, a substantial gender gap persists in medicine, particularly in academic leadership roles [1,2,3,4,5]. Women remain underrepresented among full professors and departmental chairs across specialties and countries [6].

Similarly, in Germany, despite women currently accounting for approximately 65% of all medical students and having outnumbered men among medical students since 1999 [7], female physicians remain significantly underrepresented in leadership positions within inpatient medicine. This trend is documented across numerous healthcare systems and extends far beyond German and European borders [8]. In the United States, women held only 25% of department chairs, 27% of medical school deans, and 29% of full professors in 2023 [9]. Similarly, in Canada, women accounted for just 28.1% of full professors in 2022 [10]. A striking pattern can be observed in Japan, with only 4.7% female full professors in 2019 [11]. Within Europe, a multicenter comparison across four major academic health centers (Berlin, Vienna, Oxford, and Stockholm) confirmed a similarly declining female representation along the academic career trajectory, with Sweden performing comparatively best [8]. This study also shows that gender disparities are more pronounced within university settings than in hospital environments. Additional data from Italy revealed a comparable imbalance, with only 21.9% of full professors, and more specifically 24.4% in psychiatry and 17.4% in neurology, being women [12].

The underrepresentation of women has an impact not only on the health of female physicians [13] but also on the quality of patient care [5,14]. Female physicians have a higher risk of burnout, which may be linked to gender-related differences in daily clinical work. They tend to follow guidelines more consistently, provide more preventive care, and prioritize patient-centered communication. These practices are linked to better patient outcomes but may also require more time and, therefore, increase the risk of burnout [13,14]. Literature shows that promotion disparities are profound and linked to various causes, including gender bias and limited mentoring opportunities. These disparities can delay career progression and reduce representation in leadership positions [3,4,5,15]. The inequality extends beyond career advancement itself and is also evident in the gender-based pay gap, which appears even in starting salaries and persists across specialties [16,17,18].

This study aims to systematically examine the gender gap in leadership positions in Germany and to trace their development over the past two decades, using previously established and newly modified indices. The analysis focuses on psychiatry/psychotherapy and contrasts their development with that observed in neurology. These two specialties provide an informative comparison because they are clinically and academically related fields within neurosciences and mental healthcare but differ in their clinical focus and modes of patient assessment. Psychiatry/psychotherapy was selected because it shows a comparatively strong female presence in both specialist and leadership positions within medicine [19,20]. Structured working hours, a lower emergency workload, and an emphasis on interpersonal and communicative aspects are possible reasons why psychiatry/psychotherapy appeals to many female students [20,21,22,23,24]. In contrast, neurology has historically been characterized by a more male-dominated workforce and a wider gender gap [25,26]. Comparing these two related but differently structured specialties, therefore, allows assessment of whether gender disparities in leadership follow similar patterns across neighboring disciplines or differ according to specialty-specific workforce characteristics.

The study assessed both temporal (2004–2024) and spatial dimensions, analyzing changes across 38 German medical faculties to identify regional differences, including potential disparities related to the historical East–West division of Germany. Women’s representation was evaluated across three career levels, from medical students to specialists and ultimately to leadership positions, using both the established Brüggmann-Groneberg (BG) Index and a newly developed clinical leadership index [27,28,29]. This multi-step approach enables longitudinal monitoring of career progression rather than isolated endpoint comparisons. It therefore provides a more comprehensive view of gender dynamics in medicine.

## 2. Methods

### 2.1. Study Design

This observational, retrospective study employed a nationwide, spatio-temporal approach to examine gender representation in psychiatry/psychotherapy and neurology in Germany over a 20-year period (2004 to 2024). The study combined cross-sectional data from five observation years (2004, 2009, 2014, 2019, and 2024), allowing temporal developments to be assessed at standardized five-year intervals.

**Figure 1:**
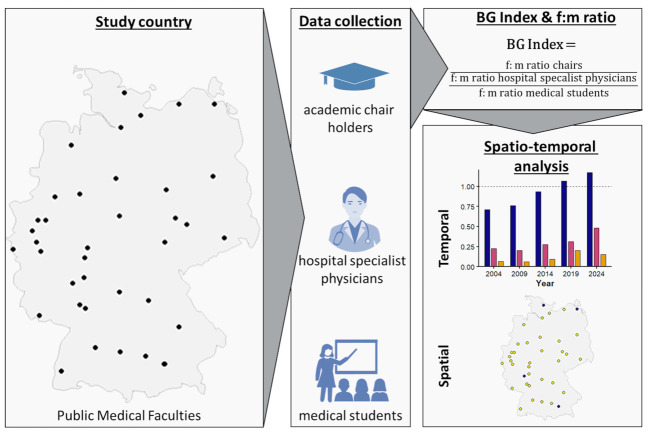
Schematic overview of the study design on gender disparities in psychiatry/psychotherapy and neurology. Dots in the map of Germany indicate the included public medical faculties. Blue dots represent sites with a female department chair, yellow dots represent sites without a female department chair. In the schematic bar chart, blue represents hospital specialists, pink represents lead physicians, and orange represents department chairs. The dashed line indicates gender parity, defined as an f:m ratio of 1.

The analysis included three main career levels: medical students as the lowest career level, hospital-based specialists as the intermediate career level, and leadership positions as the highest career level. Leadership was defined as either clinical leadership (e.g., chief physicians) or academic leadership (department chairs and professorships). The overall study design and the included career levels are illustrated in Figure 1.

### 2.2. Analyzed Career Levels

For each reference year, gender-specific data were collected for the career levels included in the analysis.

### 2.3. Medical Students

Medical students represent the entry level and lowest career stage. Data on general medical student numbers in Germany were collected to provide a national baseline for the analysis.

### 2.4. Specialist Physicians

Specialist physicians in the fields of psychiatry/psychotherapy and neurology represent the mid-career level. In this study, only hospital-based specialists were considered. Psychiatry and psychotherapy were analyzed as one combined specialty, as this corresponds to the German specialist designation and to the classification used in the physician statistics of the German Federal Chamber of Physicians (Bundesärztekammer BÄK) [19,30].

### 2.5. Academic and Clinical Leadership Positions

Leadership positions represent the highest career level and were assessed in two ways, depending on the applied index. For the academic BG Index, the highest attainable level in academic medicine was defined as chairs of university departments who simultaneously serve as heads of departments at university hospitals. For the modified clinical BG Index, leadership was defined as general clinical leadership roles, such as chief physicians, in German hospitals.

### 2.6. Data Sources

The data on general medical student numbers in Germany were obtained from the Federal Statistical Office of Germany (Statistisches Bundesamt) [7]. For each reference year, the number of medical students was documented at the beginning of the winter semester (e.g., winter semester 2004/2005 for the year 2004). The data on medical specialists were obtained from the annually published physician statistics of the Federal Chamber of Physicians [19,31,32,33,34,35]. This German institution publishes relevant data on the physician demography in Germany. The statistics also contain information on physicians in leading positions, which was extracted and subsequently applied to the later-described BGclin Index.

For the highest academic career level, all 38 public medical faculties in Germany were included. Data on department chairs were collected through official university hospital websites and supplemented by online searches (e.g., press releases, CVs).

### 2.7. Data Collection and Curation Procedures

Data collection was carried out in April 2025 and recorded in tabular form using Microsoft Excel. For each reference year and career level, the total number of persons, the number of women, the number of men, the female-to-male ratio, and the percentage of women were documented or calculated.

Because several universities no longer explicitly use the term “chair”, positions that were not referred to as such were defined for this analysis as the combination of a full or university professorship and directorship of the respective psychiatry/psychotherapy or neurology hospital department. In Germany, the position of a university chair often corresponds with that of director or head of a university hospital department [27]. For clarity, this position is hereafter referred to as department chair. The gender assignment was based on first names, titles, or publicly available photographs. A non-binary classification was not possible based on publicly available information and was therefore not considered. We recognize that this restriction does not reflect the full diversity of gender identities, and future studies should take this dimension more into consideration. For each year and each university, the gender of the department head was coded as “m” (male), “f” (female), or “0” (no eligible chair position). Provisional, acting, or vacant positions were not counted as eligible chair positions for the respective observation year and were therefore coded as “0”. Since some universities additionally have multiple or jointly held department chairs, the total number of sites included in the calculation may fluctuate around 38 from year to year, as this reflects the number of public medical faculties in Germany included in the analysis. In cases of multiple or jointly held department chairs, each formally appointed chairholder fulfilling the inclusion criteria was counted separately. In cases of regular leadership changes within a reference year, the gender of the newly appointed person was recorded. However, in cases of a change to provisional leadership, the last officially appointed person was counted.

The universities of Augsburg, Bielefeld, and Oldenburg implemented university hospitals only during the study period due to the new establishment of medical faculties (Oldenburg: 2012, Augsburg: 2016, Bielefeld: 2018) [36,37,38]. The appointment of a corresponding professorship and the assignment of clinical leadership, however, did not necessarily occur simultaneously with the establishment of the faculty. In several cases, these appointments took place later, and the respective sites were included in the analysis only once a department chair of psychiatry/psychotherapy or neurology had been formally appointed [39,40,41,42,43,44,45,46]. Before these formal appointments, the respective sites were coded as “0”, indicating that no eligible chair position existed at that time.

### 2.8. Sex Ratio

For each career level and reference year, the extracted gender data were used to calculate the female-to-male ratio (f:m) and the percentage of women in psychiatry/psychotherapy and neurology. The sex ratio over time was visualized in R (version 4.4.3) using RStudio (version 2024.12.1) and the packages ggplot2, dplyr, tidyr, and patchwork. Bar plots were generated for each specialty to depict temporal trends across career levels. Numbers above 1 indicate an f:m ratio in favor of women.

### 2.9. BG Indices

The previously established BG Index [27,28] has been constructed to track the ascension of women’s career trajectories, starting from their education as students to reaching positions as academic chairholders.

The index excludes secondary variables such as cultural background or parental status to allow for a simplified and standardized comparison of career advancement across different medical specialties. Although this study focuses on hospital-based medicine, the BG Index can also be adapted for use in outpatient settings or even nonmedical disciplines [27].

Initially, the progression from medical student to department chair is calculated by dividing the respective ratios [27,28]:

(1) $$BG\;Index=\frac{{\text{f:m}\;ratio\;chairs}_{Medical\;specialty}}{{\text{f:m}\;ratio\;medical\;students}_{Germany}}$$

An index value of 1 would indicate complete gender equity at all career levels.

The final, previously established BG Index [27,28] includes one additional career level in its calculation. By adding specialist physicians as an intermediate level, the index allows identification of the career stage at which the greatest decline in female representation occurs. If a specialty already has a low proportion of women among its physicians, leadership positions are also more likely to reflect this imbalance. Still, this can only be differentiated if the specialist level is included in the calculation. This level not only reflects the retention of women in the field but also serves as an indicator of how attractive and accessible the specialty is to female physicians [27,28]. To distinguish between physicians who find this field attractive for outpatient practice and those who pursue a hospital-based career with the potential of obtaining a chair position, only data from inpatient specialists were included.

In the following, the previously established BG Index will be referred to as the academic BG Index.

The academic BG Index was calculated as follows for each university and for each of the five time points (2004, 2009, 2014, 2019, and 2024) [27].

(2) $$Academic\;BG\;Index=\frac{\frac{{f:m\;ratio\;chairs}_{Medical\;specialty}}{{f:m\;ratio\;hospital\;specialist\;physicians}_{Medical\;specialty}}}{{\text{f:m}\;ratio\;medical\;students}_{Germany}}$$

The index focuses on the three described career levels within academic medicine: medical students (lowest career level), hospital specialists (mid-career level), and department chairs (highest career level). The earlier calculated f:m ratios at each level are set in relation to one another within this formula. It visualizes how many women reach chair positions in comparison to their initial level. The index enables temporal comparisons as well as comparisons between specializations or institutions [27]. It captures gender representation at the highest academic career level and includes approximately 35–50 chairholders. This range results from structural variation across the 38 public medical faculties, such as joint appointments or temporary vacancies.

In addition to the academic BG Index, a modified clinical version (BGclin Index) was developed. In this index, the highest career level is not defined by a small number of department chairholders but by several hundred leading positions, such as chief physicians in clinical psychiatry/psychotherapy or neurology departments in Germany. While the academic BG Index captures gender imbalance exclusively within university-based academic structures, the BGclin Index reflects gender representation within the broader clinical leadership landscape. This dual approach allows gender disparities to be analyzed not only within academic medicine but also across wider clinical career trajectories.

In detail, this BGclin Index was calculated using the f:m ratio of leading clinical positions as the numerator, including non-university department heads and chief physicians of psychiatry/psychotherapy and neurology clinical departments in German hospitals, as follows:

(3) $$BGclin\;Index=\frac{\frac{{f:m\;ratio\;leading\;positions}_{Medical\;specialty}}{{f:m\;ratio\;hospital\;specialist\;physicians}_{Medical\;specialty}}}{{\text{f:m}\;ratio\;medical\;students}_{Germany}}$$

All indices were calculated using unrounded original f:m ratios. Ratios are presented to two decimal places, whereas BG indices are reported to three decimal places to avoid loss of relevant variation.

The academic BG and BGclin indices were graphically visualized in R (version 4.4.3) using RStudio (version 2024.12.1) and the packages ggplot2, dplyr, tidyr, viridis, and grid.

### 2.10. Statistical Analysis

The analyses were descriptive and aimed to monitor gender representation across predefined career levels and observation years. Female-to-male ratios, percentages of women, and BG Indices were calculated to describe temporal and spatial patterns. No inferential statistical analyses, regression models, or formal trend tests were performed, as the objective was descriptive surveillance rather than hypothesis testing.

### 2.11. Spatial Analysis and Mapping

To analyze spatio-temporal trends across Germany, we calculated the percentage of female chairholders in psychiatry/psychotherapy and neurology for each university and year between 2004 and 2024. In most cases, the department chair is held by only one person; if a woman occupies this position, it is reflected as 100%. At universities with multiple or jointly held chairs, the percentage corresponds to the proportion of women among all chair positions, resulting in lower values (e.g., 25% when one out of four of these positions is held by a woman). These data were visualized on maps to illustrate geographic patterns and temporal changes in female representation at the level of department chairs.

University-level results were complemented by aggregated summaries at the federal and state levels, and an additional map combined both specialties to highlight overlaps and differences within the study period. All analyses and visualizations were performed in R (version 4.4.3) using RStudio (version 2024.12.1) and the packages readr, dplyr, tidyr, ggplot2, viridis, sf, rnaturalearth, rnaturalearthdata, and rgeoboundaries. Geospatial data were obtained from public sources provided by the rnaturalearth and rgeoboundaries databases, and all visualizations were generated with consistent geographic boundaries and coordinate settings to ensure comparability across years.

## 3. Results

### 3.1. Gender Distribution Among Medical Students in Germany (2004–2024)

**Figure 2:**
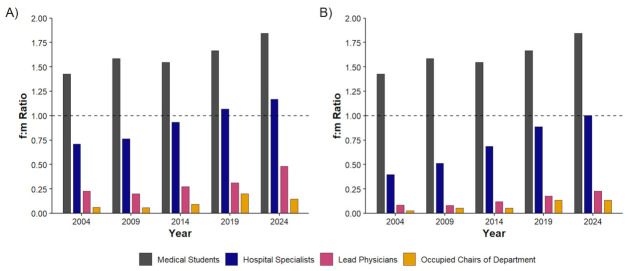
Gender representation in psychiatry/psychotherapy (**A**) and neurology (**B**) by career level (2004–2024). An f:m ratio of 1 means gender parity. Values above 1 indicate a higher proportion of women and values below 1 a higher proportion of men.

In 2004, out of 79,866 total medical students, 46,939 were women, resulting in an f:m ratio of 1.43. By 2009, this ratio had increased to 1.59 and slightly declined in 2014 to 1.55. In 2019, the total number of students reached 98,736, including 61,700 women, with an f:m ratio of 1.67. In 2024, the number of students increased further to 117,916, of whom 76,482 were women, resulting in an f:m ratio of 1.85 (Table 1, Figure 2).

### 3.2. Gender Distribution Across Specialist and Leadership Levels

The proportion of women among hospital-based specialists in psychiatry/psychotherapy increased steadily across the five reference years. In 2004, 3661 specialists were recorded, with an f:m ratio of 0.71. This ratio continued to rise, and by 2019, women outnumbered men for the first time (f:m ratio 1.07). In 2024, female representation further increased to 3621 women among 6716 specialists (f:m ratio 1.17). In contrast, a clear gap to parity remained in leading clinical positions. The f:m ratio among lead physicians was 0.23 in 2004, declined slightly to 0.20 in 2009, increased to 0.28 in 2014, and 0.31 in 2019. For 2024, the ratio rose to 0.48, with 347 women out of 1067 leadership positions. A comparable pattern was observed in neurology. The proportion of female hospital-based specialists increased continuously from an f:m ratio of 0.40 in 2004 to 1.00 in 2024, when 3180 women and 3175 men were recorded.

However, women remain markedly underrepresented in neurology’s clinical leadership roles, with ratios of 0.08 in 2004 and 2009, 0.12 in 2014, 0.18 in 2019, and 0.23 in 2024. Overall, the proportion of female specialists in psychiatry/psychotherapy increased by 12.4 percentage points, from 41.5% (1520/3661) in 2004 to 53.9% (3621/6716) in 2024, whereas neurology showed a substantially larger increase of 21.6 percentage points, from 28.4% (627/2206) in 2004 to 50.0% (3180/6355) in 2024 (Table 1, Figure 2).

### 3.3. Gender Distribution Among Department Chairs in Psychiatry/Psychotherapy and Neurology

At the level of department chairs in psychiatry/psychotherapy, female representation remained consistently low across the observation period. In 2004, 2 of 34 chairs were held by women, corresponding to an f:m ratio of 0.06. This proportion remained largely unchanged in 2009 (2 of 36 chairs; f:m ratio 0.06) and increased slightly in 2014 (3 of 36 chairs; f:m ratio 0.09). By 2019, 6 of 36 chairs were occupied by women (f:m ratio 0.20). In 2024, a slight decline was observed, with 5 women among 39 chairs (f:m ratio 0.15). A similarly pronounced gender imbalance was observed in neurology. In 2004, 1 of 38 department chairs was held by a woman (f:m ratio 0.03). In both 2009 and 2014, women occupied 2 of 39 chair positions (f:m ratio 0.05). By 2019, 5 of 42 chairs were occupied by women and 37 by men (f:m ratio 0.14). In 2024, 6 out of 50 chairs were held by women and 44 by men (ratio 0.14) (Table 1, Figure 2).

### 3.4. Temporal Trends in BG Indices in Psychiatry/Psychotherapy and Neurology

**Figure 3:**
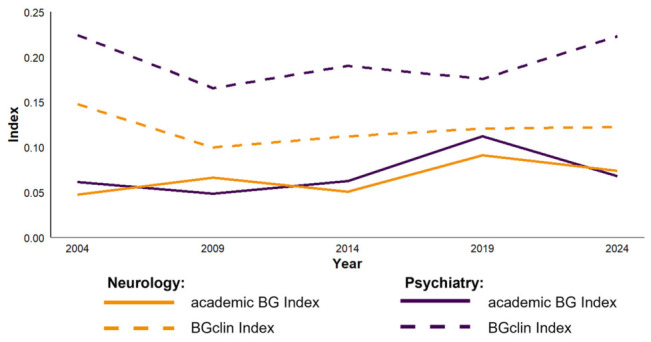
Development of academic BG Index and modified BGclin Index in psychiatry/psychotherapy and neurology (2004–2024).

In psychiatry/psychotherapy, the academic BG Index showed modest fluctuations over time. It increased from 0.062 in 2004 and peaked at 0.112 in 2019 before decreasing to 0.068 in 2024. In neurology, the academic BG Index showed a gradual rise with minor variation, reaching its highest value of 0.091 in 2019. While index values in neurology were generally comparable to those observed in psychiatry/psychotherapy, they exceeded those of psychiatry/psychotherapy in 2009 and 2024. Across both specialties, the modified BGclin Index yielded higher values than the academic BG Index throughout the study period. Psychiatry/psychotherapy consistently demonstrated higher BGclin Index values than neurology. Both specialties followed a similar temporal pattern, with an initial decline between 2004 and 2009, followed by increasing values in the subsequent years (Table 2, Figure 3).

### 3.5. Spatial Distribution of Female Department Chairs in Psychiatry/Psychotherapy and Neurology

**Figure 4:**
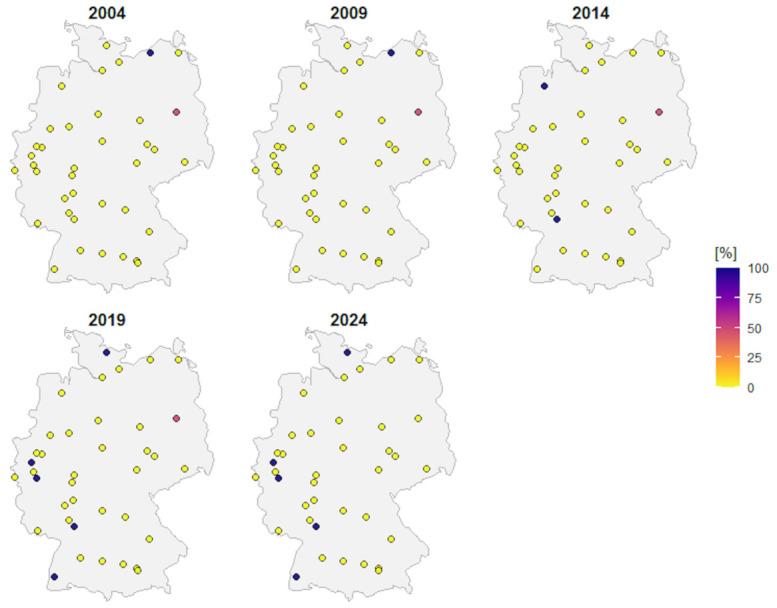
Spatial distribution of psychiatry/psychotherapy department chairs held by women across Germany, 2004–2024. Values reflect the percentage of women among all psychiatry/psychotherapy department chairs at 38 medical university sites in Germany: a single female chair corresponds to 100%, whereas shared or multi-chair structures result in values such as 50% or 25%.

In psychiatry/psychotherapy, female chairholders in 2004 and 2009 were located at Charité-University Medicine Berlin and the University of Rostock. From 2014 to 2024, additional female psychiatry/psychotherapy department chairs were observed at several universities, predominantly located in Western Germany (Figure 4).

**Figure 5:**
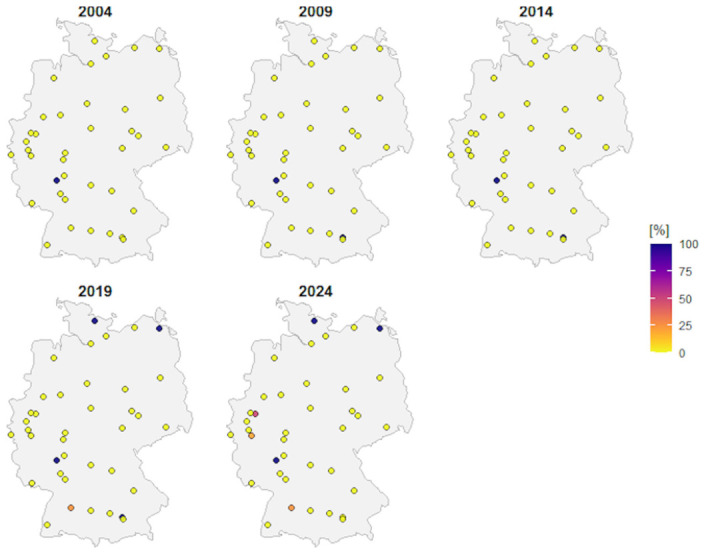
Spatial distribution of neurology department chairs held by women across Germany, 2004–2024. Values reflect the percentage of women among all neurology department chairs at 38 medical university sites in Germany: a single female chair corresponds to 100%, whereas shared or multi-chair structures result in values such as 50% or 25%.

Compared to psychiatry/psychotherapy, neurology department chairs showed even lower female representation, with only one woman in 2004 at the Johannes Gutenberg University Mainz. Overall, temporal and spatial progress was slower in neurology and remained largely confined to a few universities in Western Germany (Figure 5).

**Figure 6:**
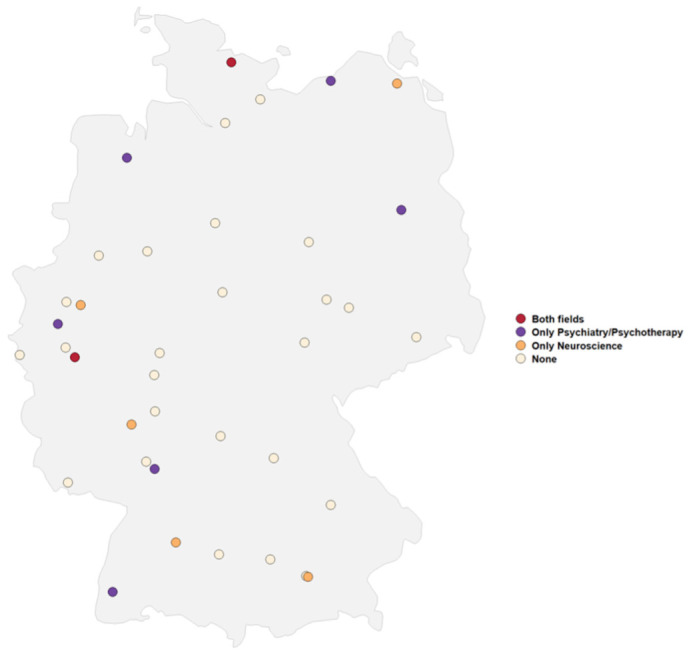
Spatial distribution of women-held psychiatry/psychotherapy and neurology department chairs across Germany, 2004–2024. Values indicate locations where women held department chairs in both fields, psychiatry/psychotherapy only, neurology only, or neither field.

Across the two specialties analyzed in Germany, approximately 66% of universities (25/38) never had a female chair between 2004 and 2024, highlighting the persistent gender gap in chairholder positions across the country (Figure 6). A particular lack of female chairholders was observed in Central-East Germany, including former East and West German regions. This pattern was reflected in the distribution of chair positions across the federal states and survey years, as shown in Supplementary Figures S1 and S2.

## 4. Discussion

The findings from this nationwide spatio-temporal analysis demonstrate that substantial gender disparities persist along the medical career trajectory in psychiatry/psychotherapy and neurology in Germany. While women now constitute the majority of medical students and gender parity has recently been achieved at the specialist level, this progress is not reflected in academic or clinical leadership positions. The imbalance is most pronounced at the level of department chairs and remains evident over the entire 20-year observation period. Spatial analyses did not show a clear pattern corresponding to the historical East–West division of Germany, but female department chairs were largely absent in Central and Eastern regions throughout the study period.

### 4.1. Spatio-Temporal Trends in Leadership Positions in Psychiatry/Psychotherapy and Neurology over a 20-Year Period

When analyzing the academic BG Index in psychiatry/psychotherapy and neurology, it becomes evident that although it fluctuates, it remains well below the ideal index value of 1 and generally persists at a low level over time. This indicates that the increasing proportion of women at earlier career stages has not translated into proportional representation at the level of university department chairs. While neurology exceeded psychiatry/psychotherapy in two observation years, the overall mean academic BG Index remains slightly higher in psychiatry/psychotherapy. However, these temporal changes should not be interpreted as direct evidence of stable structural progress. Given the limited number of university chair positions, individual appointments can substantially influence the calculated f:m ratios and BG Index values. Therefore, the observed fluctuations may partly reflect appointment dynamics at individual institutions rather than genuine systemic changes in gender representation. Accordingly, these temporal trends may be interpreted as descriptive indicators of appointment patterns rather than evidence of sustained structural change.

An interesting emerging trend is the increasing use of shared leadership models in hospital departments, which have been associated with higher workforce satisfaction and improved patient care [47,48]. In this study, this development becomes particularly evident in neurology, where the university hospitals in Bonn and Tübingen have multiple full professors jointly leading their neurology departments. Such structures may create additional opportunities for female representation in leadership, as both institutions included one female neurological director in 2024 [49,50,51]. However, such shared leadership structures may also influence temporal comparisons, as changes in the number and composition of eligible chairholders can affect f:m ratios and BG Index values independently of broader systemic change.

At the specialized physician level, psychiatry/psychotherapy has consistently shown a higher proportion of female physicians and achieved gender parity earlier than neurology. Nonetheless, neurology demonstrated a greater relative increase over the study period (+21.6% vs. +12.4%). Psychiatry/psychotherapy also continues to lead in the promotion of women into leading clinical positions, as reflected by the higher BGclin Index in 2024 (Table 2, Figure 3).

To differentiate between academic and clinical leadership pathways, the BGclin Index was applied as an additional clinical extension of the established academic BG Index. It enables the monitoring of gender inequality beyond academic medicine, as it is not limited to university hospitals and the associated academic leadership positions, such as department chairs in psychiatry/psychotherapy and neurology, but also includes clinical leadership positions in non-university hospitals. The BGclin Index therefore complements existing indicators of gender equity by assessing gender disparities outside the academic setting. It allows comparison of whether gender inequalities are similarly pronounced in broader clinical leadership positions as they are in university-based academic leadership. In the present study, BGclin Index values were consistently higher than academic BG Index values, indicating that gender disparities were less pronounced in broader clinical leadership than in department chairs. Since the academic BG Index has already been used to assess gender representation in several medical specialties [27,28], the BGclin Index may likewise be applicable to further specialties and healthcare settings. However, as the BGclin Index is still a relatively new methodological extension, further application and validation in other specialties, institutions, and healthcare systems would provide a stronger basis for comparison.

As the number of female medical students continues to rise, one would expect that this trend will be reflected proportionally in the medical workforce. Although the number of female specialist physicians has increased as well, their proportion still does not match that among medical students and remains particularly low in higher leadership positions. This indicates that demographic change alone is unlikely to close the gender gap without addressing structural barriers, which contribute to women contemplating leaving academic medicine [1]. Even a growing pool of qualified female graduates cannot achieve greater parity if these underlying causes of unequal career advancement remain unsolved [52].

The spatial analysis further showed that female chairholders were clustered at a few institutions, indicating that widespread structural change has not yet occurred. This spatial perspective also illustrates that across the country, psychiatry/psychotherapy holds a higher representation of female chairholders compared to neurology (Figure 4 and Figure 5). Although more female chairholders were observed in Western Germany in recent years, this should not be interpreted as clear evidence of regional disparities. Western Germany covers a larger geographical area, includes more medical universities, and has a larger population, resulting in more institutions where women could potentially hold leadership positions. Moreover, previous research in German otolaryngology (head and neck surgery) found no clear regional differences between former Western and Eastern German states [53]. Therefore, the observed spatial pattern may rather reflect institutional distribution than historically determined regional differences.

### 4.2. Persistent Gender Patterns in Academic Medicine

The central finding of this study is consistent with the well-documented “leaky pipeline” phenomenon in academic medicine, whereby the representation of women progressively declines at higher career levels, particularly during the transition to senior leadership positions [12,54,55].

The findings of this study are supported by earlier research. Despite the feminization of medical education in Germany since the late 1990s [7], a sharp decline in female representation from medical school to senior and leadership positions has been documented. This gap intensifies along the career path [2,3,4,5,20,52,56]. This pattern is not unique to Germany but mirrors trends observed across other European countries and worldwide, where women now constitute the majority of medical students but remain markedly underrepresented in leadership roles [8,9,10,12,57].

The academic BG Index was developed as a tool to quantify gender disparities in academic medicine, revealing significant gaps between specialist and chair levels in obstetrics/gynecology and in ear, nose, and throat medicine [27]. The index has also been applied in the field of urology, where it identified pronounced leadership gaps [28]. In the previous studies, the assessment time points were also set at five-year intervals. However, because the specific survey years differed between specialties, the BG values cannot be directly compared. Nevertheless, for the overlapping period of 2013–2015, the calculated BG Indices show an increase in the following order: obstetrics/gynecology < neurology < psychiatry/psychotherapy < urology < ear, nose, and throat medicine.

When looking at gender representation across specialties, the German Medical Women’s Association (Deutscher Ärztinnenbund e.V.) has been documenting the role of women in leadership positions in academic medicine since 2016, with findings regularly published in the Medical Women on Top report series [58]. The most recent report (2024) shows that, across all medical specialties, women now make up 41% of senior physicians. However, they are still significantly underrepresented in hospital leadership, holding only 14% of clinical directorships. In the mental health sector, female clinical directors are somewhat more frequent (24%) and above the national average, while in neurology the proportion remains substantially lower (15%) [20]. These specialty-specific trends are in line with our findings, which also show higher female representation in leadership positions in psychiatry/psychotherapy than in neurology. By additionally applying the academic BG Index and the BGclin Index, our study further demonstrates that the underrepresentation of women is particularly pronounced in university-based academic leadership at the level of department chairs, especially when compared with broader clinical leadership settings.

### 4.3. Underlying Factors Contributing to the Observed Gender Trends

The observed gender differences in higher career levels may in part be explained by the structural and cultural characteristics of the two analyzed specialties. Psychiatry/psychotherapy typically offers more regular working hours, fewer emergency duties, and a stronger emphasis on interpersonal interaction, making it more compatible with work–life balance. This is a factor often prioritized by female physicians, particularly regarding family-related responsibilities [21,22,23,59]. Furthermore, gender norms may influence the choice of specialty, as women are more frequently expected to demonstrate empathy and communication skills, traits that align closely with psychiatry/psychotherapy [13,24,60]. Studies suggest that women tend to place greater value on the interpersonal dimensions of care, while at the same time technical, surgical, or research-oriented fields such as neurology continue to attract more men [59,61,62,63].

Despite growing female representation in medicine, various structural, cultural, and personal barriers continue to hinder the advancement of female physicians [15,64,65]. Among these barriers, the persistent “glass ceiling” limits access to leadership and decision-making roles [4]. External barriers include the limited access to mentorship and role models [66], harassment, gender bias [67], and the double burden of family and professional responsibilities [64]. Internal factors such as lack of self-confidence [15,65,68] and reluctance to negotiate [68,69] further limit career progression. These factors contribute to higher rates of emotional exhaustion and burnout, lower job satisfaction, and reduced opportunities for leadership, academic advancement, and equal pay [56,70,71,72].

Gender-based salary disparities may further reinforce these inequalities. Previous studies have shown that female physicians earn less than their male colleagues, even after adjusting for factors such as age, specialization, experience, and publication record [17,73,74]. Pay gaps may also differ between specialties, with the highest gap in surgical fields and narrower gaps in primary care [17,75]. Additionally, a negative correlation has been observed between the proportion of women in a specialty and its average income: as more women enter a field, average earnings tend to decline [70,76,77].

Research productivity, especially publication output, plays a central role in academic promotion and salary progression. Since publications are a key criterion for professorial appointments, this directly contributes to the persistent leadership gap [78,79,80]. Nonetheless, gender imbalances persist in scientific publishing, with male authors more frequently listed as first or senior authors, publishing more often in prestigious journals, and receiving more citations [79,81,82]. In neuroscience, female authorship rates are lowest in neurosurgery, then in neurology, and highest in psychiatry/psychotherapy [83].

Together, these structural factors may help explain why increasing female representation at earlier career stages does not automatically translate into proportional representation in senior academic or clinical leadership positions.

### 4.4. Limitations of the Study

Several limitations should be considered when interpreting the findings.

First, the BG Index is a relative measure and sensitive to changes in the f:m ratio among medical students. An increasing denominator can lower the index even if the representation of women in leadership positions remains unchanged.

Second, the small sample size at the level of department chairs introduces instability, as individual appointments can substantially influence f:m ratios and BG Index values. Observed increases or declines between observation years may therefore reflect individual appointment dynamics or random variation rather than stable structural progress or true regression. Nevertheless, the academic BG Index remains useful for identifying overarching trends in gender representation despite short-term fluctuations.

The spatial analysis should also be interpreted with caution. Since most universities have only one department chair in psychiatry/psychotherapy or neurology, a single female appointment results in 100% representation, potentially overstating gender equality. Thus, the map is better suited to illustrating structural patterns and emerging trends rather than measuring sustained progress.

Further limitations include the use of a binary gender classification based on publicly available data, including first names, titles, and photographs. This approach does not capture the full diversity of gender identities.

Moreover, the BG Index does not account for confounding variables, including part-time work, parental leave, years of professional experience, or differences in research productivity. Institutional conditions that may influence career advancement, such as local appointment procedures, mentoring opportunities, family-supportive structures, or gender equality measures, could not be systematically assessed.

Finally, leadership data were obtained mainly from university websites and other publicly available sources. These sources may contain incomplete, outdated, or inconsistently presented information. Although all available information was carefully checked, some inaccuracies cannot be fully excluded. Moreover, there may be variation between sources and universities in how leadership structures, academic positions, and clinical responsibilities are presented.

Due to the nationwide observational and descriptive study design, this study can identify temporal and spatial patterns of gender representation but cannot determine the causal mechanism underlying the observed disparities.

Despite these limitations, the present findings of our study offer valuable insights into ongoing gender disparities across career levels and in academic and clinical leadership roles. They highlight the need for continued efforts to promote equity in psychiatry/psychotherapy and neurology. By applying both the academic BG Index and the modified BGclin Index, we compared leadership pathways and gender imbalances both within academic structures and in wider clinical leadership positions not limited to university settings. Our results provide reliable and objective data that can inform equality strategies, improve appointment processes, and guide the development of measures to advance women in academic medicine. Through this approach, our research contributes meaningfully to promoting gender equity in medical leadership sectors that remain predominantly male-dominated.

### 4.5. Research Implications

Our temporal analysis demonstrated a clear gender gradient, with women well represented in early career stages but persistently underrepresented in leadership roles in psychiatry/psychotherapy and neurology. The longitudinal design enabled identification of trends over time that would not be apparent in single-time-point analyses. Our spatial analysis showed that female chair representation is concentrated at a limited number of universities, highlighting the decisive role of institutional factors and the need for site-specific reforms. The combined use of the academic BG Index and the clinical BG Index illustrates existing differences between academic and clinical leadership positions and provides a transferable framework for comparisons across specialties, institutions, and professional fields, supporting future structural reforms.

As the BG Indices provide exclusively quantitative insights, future research should complement them with qualitative approaches. Including the experiences and perspectives of women in leadership pathways and considering intersectional factors such as age, parenthood, part-time employment, and other sociodemographic characteristics may help identify dimensions of gender equity that are not fully captured by quantitative measures.

### 4.6. Practical Implications and Recommendations

Despite recent gains in gender representation at earlier career levels, substantial gender disparities persist at higher levels of academic and clinical leadership. Addressing these inequities requires systemic change, including transparent salary structures [73,75] and accessible family-friendly working conditions [71,84]. Structured mentoring [1,66] and leadership development programs [74,84] tailored to women’s advancement, along with equalized starting salaries [16] and negotiation training [68,69], are further essential measures for addressing persistent disparities. Promoting leadership diversity is not only a matter of equity but also contributes to better scientific quality and improved clinical outcomes, productivity, and team dynamics [83,85,86]. The implementation and effectiveness of these measures can be systematically monitored using standardized tools such as the academic BG and clinical BG Indices, allowing longitudinal evaluation of progress and identification of remaining structural gaps.

## 5. Conclusions

In this 20-year spatio-temporal analysis, we demonstrate that, despite the increasing feminization of medical education in Germany, substantial gender disparities persist in leadership positions within psychiatry/psychotherapy and neurology. While gender parity has been achieved at the level of hospital-based specialists, women remain markedly underrepresented among department chairs and clinical leaders. The academic BG Index and the modified BGclin Index reveal that these disparities are enduring, with progress slower in neurology than in psychiatry/psychotherapy. Spatial analyses further showed that female chair positions were concentrated in a small number of institutions, with persistent gaps in Central and Eastern Germany. Overall, demographic change alone has not sufficed to overcome structural barriers, highlighting the persistence of the “leaky pipeline” in academic and clinical medicine.

## Figures and Tables

**Table 1:** Gender distribution across career levels in psychiatry/psychotherapy and neurology in Germany, 2004–2024.

Year	Variable	Medical Students	Psychiatry and Psychotherapy			Neurology		
			Hospital Specialists	Lead Physician	Occupied University Chairs	Hospital Specialists	Lead Physician	Occupied University Chairs
2004	Total	79,866	3661	557	34	2206	388	38
	Female	46,939	1520	103	2	627	30	1
	Male	32,927	2141	454	32	1579	358	37
	f:m ratio	1.43	0.71	0.23	0.06	0.40	0.08	0.03
	% Female	58.8%	41.5%	18.5%	5.9%	28.4%	7.7%	2.6%
2009	Total	79,929	4432	642	36	3112	440	39
	Female	49,022	1917	107	2	1054	33	2
	Male	30,907	2515	535	34	2058	407	37
	f:m ratio	1.59	0.76	0.20	0.06	0.51	0.08	0.05
	% Female	61.3%	43.3%	16.7%	5.6%	33.9%	7.5%	5.1%
2014	Total	87,863	5294	820	36	4133	612	39
	Female	53,352	2557	177	3	1682	65	2
	Male	34,511	2737	643	33	2451	547	37
	f:m ratio	1.55	0.93	0.28	0.09	0.69	0.12	0.05
	% Female	60.7%	48.3%	21.6%	8.3%	40.7%	10.6%	5.1%
2019	Total	98,736	6079	939	36	5266	699	42
	Female	61,700	3140	224	6	2475	106	5
	Male	37,036	2939	715	30	2791	593	37
	f:m ratio	1.67	1.07	0.31	0.20	0.89	0.18	0.14
	% Female	62.5%	51.7%	23.9%	16.7%	47.0%	15.2%	11.9%
2024	Total	117,916	6716	1067	39	6355	784	50
	Female	76,482	3621	347	5	3180	145	6
	Male	41,434	3095	720	34	3175	639	44
	f:m ratio	1.85	1.17	0.48	0.15	1.00	0.23	0.14
	% Female	64.9%	53.9%	32.5%	12.8%	50.0%	18.5%	12.0%

Career levels include medical students, hospital-based specialists, lead physicians, and university chairs in Germany. Abbreviations: f:m, female-to-male ratio.

**Table 2:** Academic BG Index and modified BGclin Index for psychiatry/psychotherapy and neurology (2004–2024).

Year	Psychiatry and Psychotherapy		Neurology	
	Academic BG Index	Modified BGclin Index	Academic BG Index	Modified BGclin Index
2004	0.062	0.224	0.048	0.148
2009	0.049	0.165	0.067	0.100
2014	0.063	0.191	0.051	0.112
2019	0.112	0.176	0.091	0.121
2024	0.068	0.223	0.074	0.123

Abbreviations: BG Index: Brüggmann-Groneberg Index, BGclin Index: clinical Brüggmann-Groneberg Index.

## Data Availability

All relevant data are included within the manuscript and were obtained from publicly available sources.

## Supplementary Materials

The following supporting information can be downloaded at: https://www.mdpi.com/article/10.3390/jcm15145608/s1. Figure S1. Female psychiatry/psychotherapy chairholders across German federal states, 2004–2024. Color intensity represents the number of university sites with female chairholders per state and survey year. Figure S2. Female neurology chairholders across German federal states, 2004–2024. Color intensity represents the number of university sites with female chairholders per state and survey year.

## Abbreviations

BG Index	Brüggmann-Groneberg Index
BGclin Index	clinical Brüggmann-Groneberg Index
F:m ratio	female-to-male ratio
