# Supplementary material for: A 20-Year Spatio-Temporal Analysis of Gender Workforce in Medical Departments of Psychiatry/Psychotherapy and Neurology in Germany
Source: J Clin Med. 2026 Jul 17;15(14):5608. doi: 10.3390/jcm15145608 (PMC13412614; doi:10.3390/jcm15145608)
Supplement: Supplementary file 1 [file jcm-15-05608-s001.zip › jcm-4411723-supplementary.pdf]

Supplementary Materials

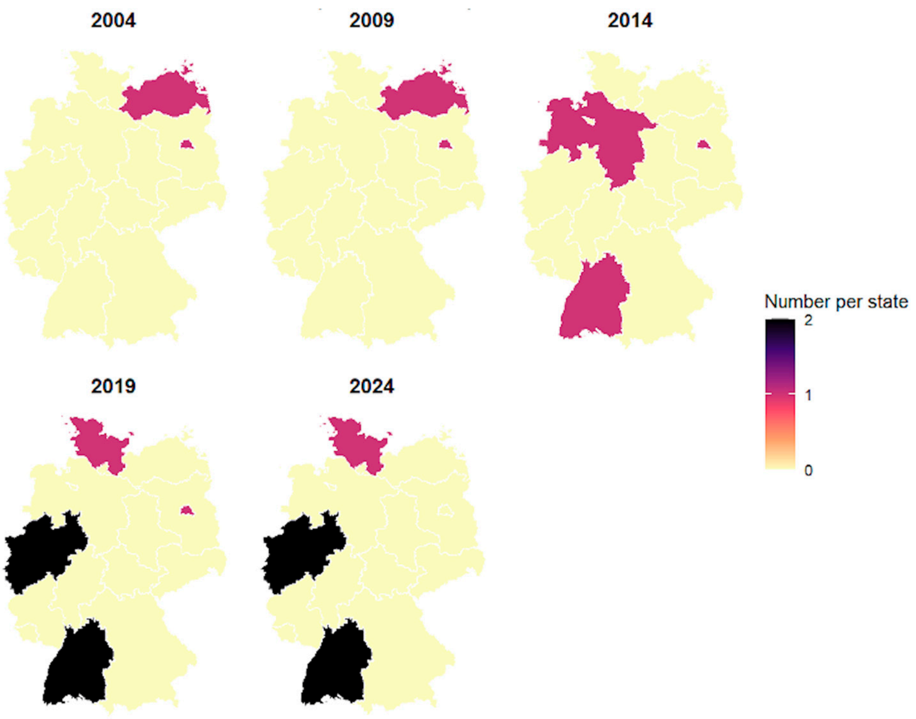

Figure S1. Female psychiatry/psychotherapy chairholders across German federal states, 2004-2024. *Color intensity represents the number of university sites with female chairholders per state and survey year.*

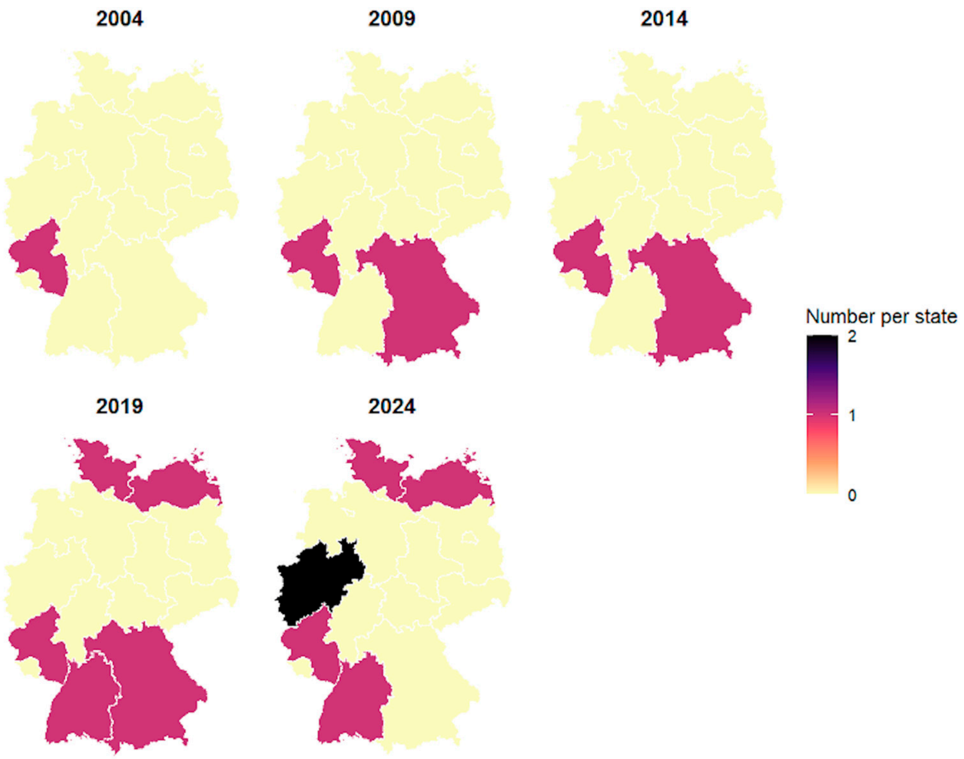

Figure S2. Female neurology chairholders across German federal states, 2004-2024. *Color intensity represents the number of university sites with female chairholders per state and survey year.*
